# A case of anti-neutrophil cytoplasmic antibody-associated vasculitis with anti-glomerular basement membrane antibodies that was successfully treated with mizoribine as a safe and effective remission maintenance therapy with prednisolone and plasma exchange

**DOI:** 10.1007/s13730-019-00423-2

**Published:** 2019-10-14

**Authors:** Yuki Ikeda, Kenichi Fukunari, Saori Uchiumi, Yuki Awanami, Akiko Kanaya, Keiichiro Matsumoto, Makoto Fukuda, Tsuyoshi Takashima, Motoaki Miyazono, Yuji Ikeda

**Affiliations:** 1 Department of Nephrology, Sasebo Kyosai Medical Center, 10-17 Shimanjityo, Sasebo, Nagasaki, 857-8575 Japan; 2 Department of Internal Medicine, Faculty of Medicine, Saga University, Saga, Japan

**Keywords:** Anti-glomerular basement membrane (anti-GBM) antibody, Anti-neutrophil cytoplasmic antibody (ANCA), Mizoribine, Hemodialysis

## Abstract

We herein report the case of myeloperoxidase (MPO) anti-neutrophil cytoplasmic antibody (ANCA)-associated vasculitis with anti-glomerular basement membrane (anti-GBM) antibody positivity that successfully treated with mizoribine (MZR) as an immunosuppressive drug for remission maintenance therapy after the initiation of dialysis in addition to plasma exchange (PE) and glucocorticoid treatment to control the disease condition. A 79-year-old woman developed serious renal dysfunction and pulmonary alveolar hemorrhaging due to MPO–ANCA and anti-GBM antibody double-positive vasculitis. She was started on hemodialysis and was treated with methylprednisolone (m-PSL) pulse therapy with PE, followed by oral prednisolone (PSL). The pulmonary alveolar hemorrhaging disappeared, and both antibody titers immediately decreased but then rose again. Thus, m-PSL pulse therapy performed again in combination with combined with MZR treatment. Her poor renal function was irreversible; however, this therapy decreased both antibody titers, and they did not increase again. The patient developed pancytopenia and hyperuricemia. It was considered likely that these conditions developed in association with MZR treatment. We, therefore, measured the patient’s blood concentration of MZR, and the maintenance dose was finally set at 50 mg after each dialysis session. The patient’s pancytopenia and hyperuricemia improved and PSL could be smoothly tapered. This is the first case report of the use of MZR for remission maintenance therapy in a patient on hemodialysis who was positive for both ANCA and anti-GBM antibodies. The findings suggest that MZR can be used safely and effectively in such cases.

## Introduction

As the initial treatment of anti-neutrophil cytoplasmic antibody (ANCA)-associated vasculitis (AAV) with anti-glomerular basement membrane (anti-GBM) antibody positivity, many patients receive plasma exchange (PE) with glucocorticoid (GC) and cyclophosphamide (CYA) combination therapy in the acute phase of the disease to treat anti-GBM antibody-type rapidly progressive glomerulonephritis. For subsequent maintenance therapy, immunosuppressive drugs, such as azathioprine (AZA), mycophenolate mofetil (MMF), and methotrexate (MTX), are administered [1]. However, immunosuppressive drugs are associated with a high risk of serious adverse events, such as pancytopenia or infection, in patients with renal failure and elderly patients.

We herein report a case of myeloperoxidase (MPO)-ANCA-associated vasculitis with anti-GBM antibody positivity that was successfully treated with mizoribine (MZR) as an immunosuppressive drug for remission maintenance therapy after the initiation of dialysis in addition to PE and GC treatment to control the disease condition. The patient did not experience any serious adverse events, and the patient’s blood levels of MZR were monitored throughout the clinical course.

## Case report

The patient was a 79-year-old Japanese woman who had received medical treatment from her local doctor for hyperlipidemia and hypertension. Her renal function had been normal until 1 year previously. Gross hematuria appeared 4 days before her presentation to a local clinician, and general fatigue appeared 2 days before her presentation. She was found to have anemia and severe renal dysfunction (serum creatinine: 10.78 mg/dL) and was transferred to our hospital in December 2016. She was treated for asthma using Breo Ellipta as an inhalant and had suffered from interstitial pneumonia for several years, but showed no tendency toward exacerbation. Her family history, life history, and allergy history were unremarkable.

On admission, her height was 142.1 cm, and her body weight was 51.2 kg. Her vital signs were as follows: body temperature, 36.7 °C; blood pressure, 209/104 mmHg; pulse rate, 102 beats/min and regular; respiratory rate, 20 breaths/min; and SpO_2_, 97% (on room air). The palpebral conjunctiva showed slight pallor. Fine crackles were heard in both lower lung fields, and pitting edema was observed in both legs.

The laboratory data showed inflammation (CRP, 6.69 mg/dL) without leukocytosis (white blood cell count, 8910/μL), normocytic anemia (serum hemoglobin, 7.6 g/dL), serious renal dysfunction (serum blood urea nitrogen, 82.1 mg/dL; creatinine, 11.27 mg/dL), and massive urinary protein (UP/UC, 13.71 g/gCr) with a large number of poikilocytes. The serum MPO-ANCA and anti-GBM antibody levels were both elevated to 609 EU/mL and 19.6 EU/mL, respectively. In addition, her serum was positive for antinuclear antibodies (640 times), especially anti-centromere antibodies (elevated to 10.7 U/mL), but she showed no symptoms such as Raynaud’s phenomenon or calloused skin to suggest scleroderma. In addition, her serum KL-6 and SP-D levels were elevated to 1069 U/mL and 175.4 ng/mL, respectively, suggesting interstitial pneumonia. The detailed laboratory data on admission are shown in Table 1.

**Table 1:** Laboratory findings on admission

Peripheral blood		Blood chemistry		Serology	
WBC	8910/μL	TP	6.9 g/dL	CRP	6.69 mg/dL
Neu	84.1%	Alb	3.2 g/dL	IgG	1480 mg/dL
Lympho	12.0%	T-Bil	0.22 mg/dL	IgA	354 mg/dL
Mono	1.9%	AST	11 U/L	IgM	134 mg/dL
Eosino	0.1%	ALT	5 U/L	MPO-ANCA	609 EU
RBC	259 × 10^4^/μL	LDH	267 1U/L	Anti-GBM antibody	19.6 EU
Hb	7.6 g/dL	CPK	71 U/L	ANA	640 times
Ht	22.8%	Uric acid	8.00 mg/dL	Anti-centromere antibody	10.7 U/mL
PIT	18.0 × 10^4^/μL	BUN	82.1 mg/dL	KL-6	1069 U/mL
		Cr	11.27 mg/dL	SP-D	175.4 ng/mL
		Na	139 mEq/L		
		K	4.99 mEq/L		
		Cl	111.1 mEq/L		
*Urinalysis*		Ca	8.3 mg/dL	*Blood gas analysis*	
Protein	3+	IP	8.9 mg/dL	PH	7.206
Occult blood	3+	Blood sugar	130 mg/dL	pCO_2_	32.2 mmHg
Sugar sediment	2+	HbAlc	4.8%	HCO_3_^−^	12.3 mmol/L
RBC	> 100 HPF dysmorphic			B.E	− 14.6
WBC	10–19 HPF			Anion gap	16.2 mEq/L
Urine chemistry					
NAG	22.5 U/L				
P/C ratio	13.71 g/gCr				
p2MG	102,860 ng/mL				

**Fig. 1:**
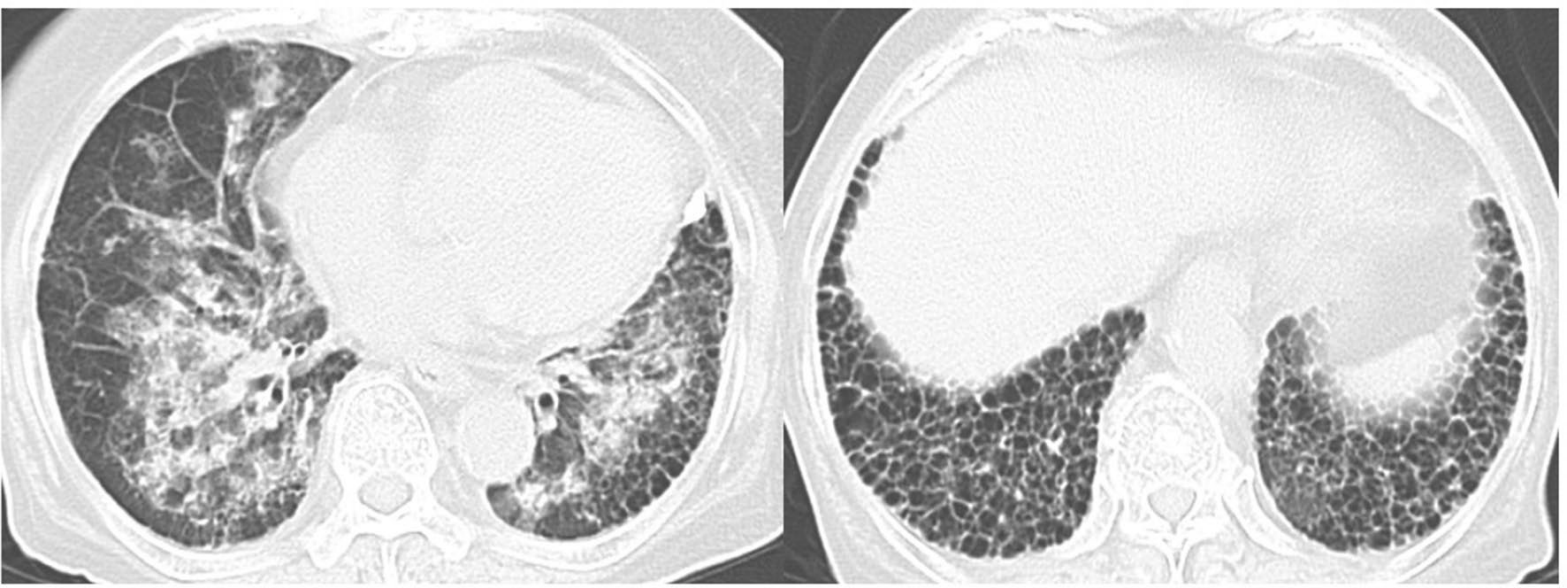
Computed tomography images of the lungs

Plain chest computed tomography (CT) showed a honeycomb pattern and slight linear reticulation at the base of both lungs. It was a usual interstitial pneumonia pattern. CT also showed infiltrative shadow and a frosted glass shadow that spread diffusely throughout the whole lung field, which was not a typical finding of interstitial pneumonia (Fig. 1). Although we could not deny that these shadows indicated pneumonia, we considered them to reflect pulmonary alveolar hemorrhage due to the presence of blood in the sputum.

Based on these findings, we diagnosed the patient with AAV with anti-GBM antibody. There were no signs or symptoms of vasculitis except in the kidneys and lungs. Her Birmingham Vasculitis Activity Score was 18 points.

The patient was dialyzed from day 2 and was treated methylprednisolone (m-PSL) pulse therapy at dose of 1 g/day with PE (FFP 40 U) for 3 days starting the same day, followed by oral prednisolone (PSL) at a dose of 40 mg/day. With regard to the PE content, we used OP-02 W (Asahi KASEI) as a dialysis membrane, and used nafamostat mesylate to prevent the coagulation of the perfusion blood (initial dose, 0 mg; sustained dose, 25 mg/h). We performed daily plasma exchange for 3 days using FFP (40 Units/time as a substitute liquid), blood flow, 150 cc/min; separated plasma flow, 50 cc/min.

**Fig. 2:**
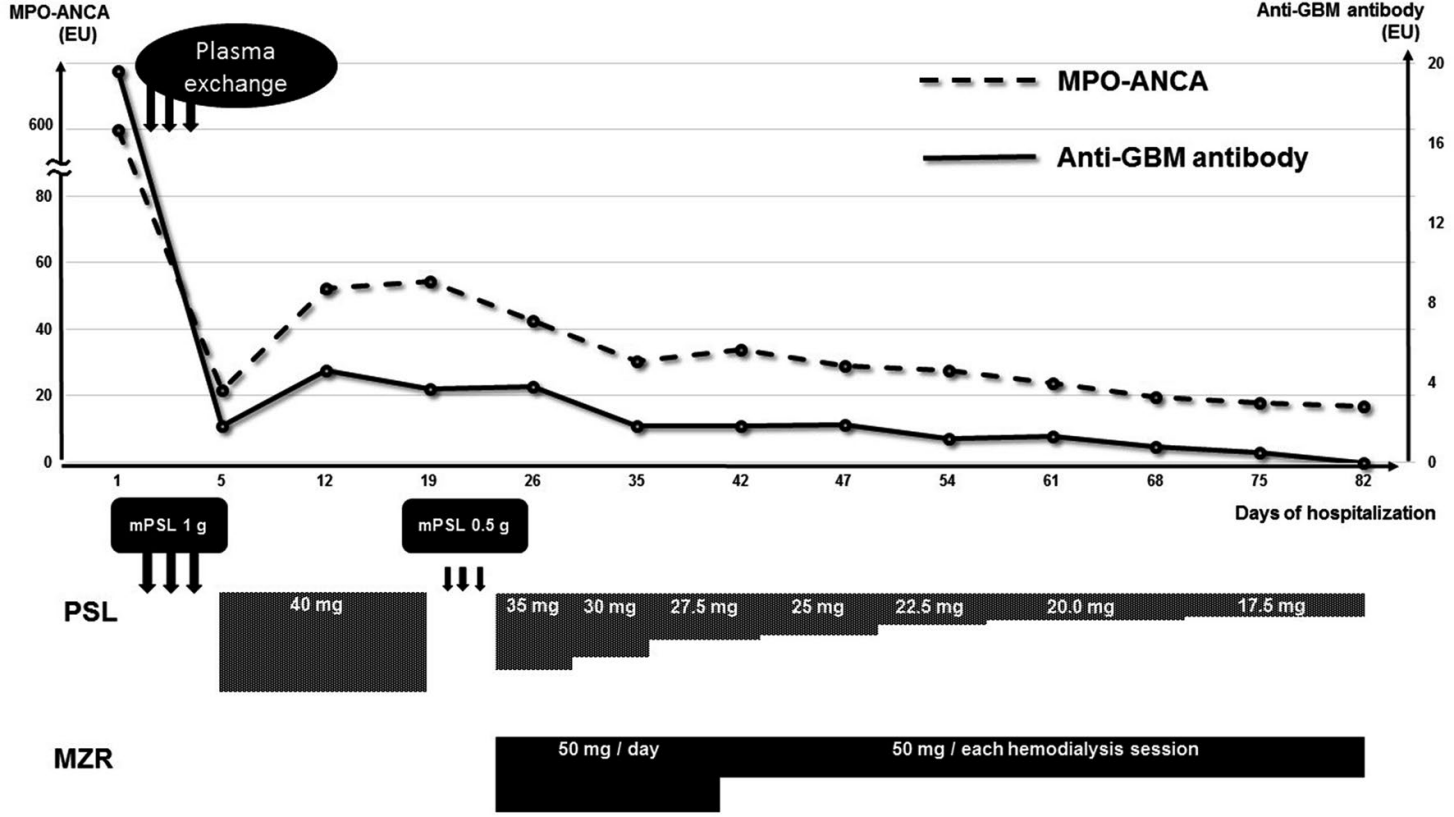
Clinical course

Both antibody titers decreased immediately but rose again from day 12. Thus, the patient received m-PSL pulse therapy at a dose of 0.5 g/day for 3 days again from day 20, with MZR (50 mg/day) added from day 23. In previous reports, MZR was only administered for patients who continued hemodialysis for several years with repeated recurrence of ANCA-related vasculitis [2, 3]. However, in the present case, it was administered at the induction of hemodialysis, and her daily urine flow was still 300–500 cc. We, therefore, set the dose to 50 mg per day. These therapies reduced the levels of both antibodies, and they did not increase again (Fig. 2).

**Fig. 3:**
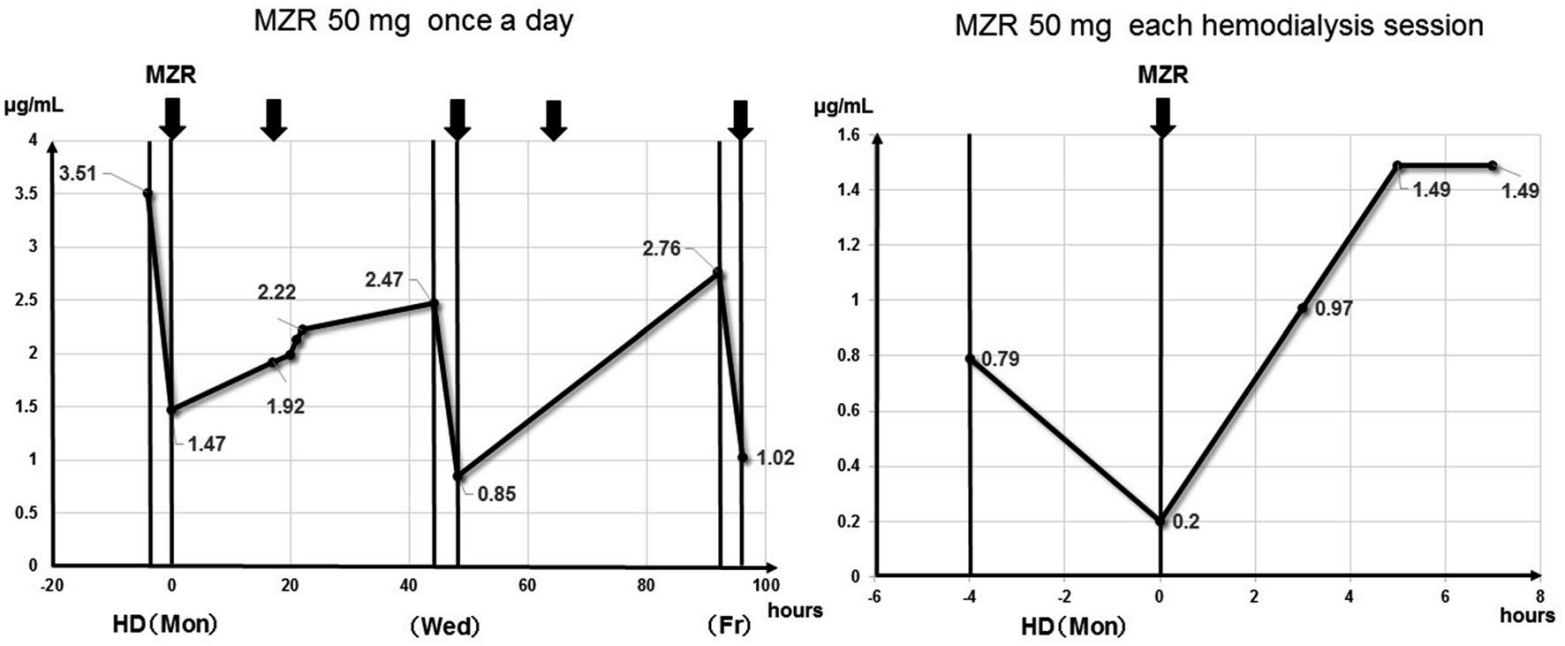
Blood concentration of mizoribine

The patient developed pancytopenia and hyperuricemia, likely as adverse events of MZR, from day 28. We, therefore, measured the patient’s blood concentration of MZR at 3, 4, and 5 h after administration (C3, C4, and C5) during hemodialysis (HD) (Fig. 3). When MZR was administered daily, the blood concentration increased over time and over the weekend. The maximum blood concentration (Cmax) was 3.51 μg/mL before dialysis at the beginning of the week. Because the concentration was not so high, we suspected sulfamethoxazole trimethoprim (used to prevent pneumocystis) to be the most likely source of the adverse event and stopped its administration. However, the pancytopenia and hyperuricemia did not improve after the cessation of this drug; thus, we considered MZR to be the cause of these adverse events and reduced the dosage. The maintenance dose of MZR was ultimately set at 50 mg after each dialysis session. Thereafter, the pancytopenia and hyperuricemia improved.

The maximum blood concentration when MZR was administered at a dose of 50 mg per dialysis session was 1.49 μg/mL (Fig. 3). Despite reducing the dosage of MZR, both antibody titers continued to decrease, and PSL could be smoothly tapered. Although pancytopenia and hyperuricemia developed as side effects, the severity was mild. The minimum of WBC count, Hb level, and Plt count were as follows: 3250/μL (no grade), 8.9 g/dl (Grade 1), and 57,000/μL (Grade 2), and there was no signs of gouty attack or other physiological abnormalities, although the maximum UA level was > 10 mg/dl (Grade 1; National Cancer Institute Common Terminology Criteria for Adverse Events v3.0) [4]. We did not perform a renal biopsy, because the patient presented with dyspnea in the acute phase. She already had severe renal dysfunction and her kidneys seemed to be atrophic on ultrasonography and computed tomography. She also suffered from heparin-induced thrombocytopenia from day 19. We were unable to confirm the linear deposition of IgG to the glomerular basement membrane by a renal biopsy in this case, so we did not prove directly that the elevated titer of anti-GBM antibody in her blood test was associated with a disease condition. There is also a possibility that elevated level of anti-GBM antibody was associated with hypergammopathy. However, we cannot exclude the possibility that the elevated titer of the anti-GBM antibody was not related to the pathogenicity of the disease, as the titer was low.

## Discussion

Cases involving patients who are positive for both ANCA and anti-GBM antibody (double-positive) are believed to be relatively rare. However, double-positive cases have been reported to account for 9.36% of patients with AAV [5]. The prognosis of double-positive cases is debatable [1, 6, 7]. McAdoo et al. retrospectively analyzed the clinical features and long-term outcomes of a large cohort of 568 AAV patients from 4 European centers, including 41 patients with anti-GBM antibody disease and 37 double-positive patients, and reported that among the double-positive patients, older patients and patients with a longer duration of symptoms before the diagnosis were likely to have AAV (age distribution showed one peak [60–75 years], and symptom duration was longer than 10–12 weeks). However, in the acute phase, the clinical course of the double-positive group closely resembles that of anti-GBM antibody disease, which is associated with serious renal failure and pulmonary alveolar hemorrhaging in approximately one-third of patients. The present case shared some similarities with respect to advanced age and complication with pulmonary alveolar hemorrhage in the acute phase.

It has also been reported that the anti-GBM antibody titers of double-positive patients tend to be lower in comparison with patients who are positive for anti-GBM antibody alone, and the renal survival of the double-positive group was shown to be better than that of patients positive for anti-GBM antibody alone and not better than in the AAV group; however, there were no marked differences in the overall survival of the groups. With respect to the treatment of the double-positive group, as mentioned above, many cases in the acute phase received PE followed by combination therapy with GC and CYA, and immunosuppressive drugs (e.g., AZA, MMF, and MTX) were often administered as maintenance therapy (74% of double-positive patients were reportedly receiving immunosuppressive drugs at 6 months). However, approximately half of double-positive patients relapsed within the 4.8-year follow-up period, and the majority of patients were not receiving maintenance treatment with immunosuppressive drugs [1].

The prolonged use of immunosuppressive drugs such as CYA or AZA is associated with a high risk of pancytopenia and severe infection; thus, physicians hesitate to prescribe these drugs, especially to elderly patients. All immunosuppressive drugs, including MZR, are associated with a risk of infection due to the side effect of myelosuppression. Furthermore, their dosage must be reduced in patients with CKD [8]. To administer these drugs safely, it is important to—when possible—measure their blood levels, and to cease their administration if the blood level becomes high. The blood level of MZR can be measured and the drug is eliminated by dialysis; thus, it is relatively easy to control the blood level. For this reason, MZR we be used more safely in comparison with other immunosuppressive drugs (e.g., CYA or AZA).

In the present case, although the patient had received PE in addition to GC therapy in the early phase, which controlled the pulmonary alveolar hemorrhaging, both the ANCA and anti-GBM antibody titers rose again. However, we were able to decrease both antibody titers again by adding MZR as an immunosuppressive drug without severe adverse events by monitoring the blood concentration of MZR.

MZR is an inhibitor of nucleic acid synthesis and has effects similar to AZA and MMF [9]. Dialysis is the only metabolic pathway for this drug in anuric patients on hemodialysis, because MZR is mainly eliminated via urine [10]. A mean of 43% of the drug was reported to be removed by 4-h dialysis [11]. While the concentration at 3 h after the administration of MZR is generally equivalent to the Cmax in patients with a normal kidney function and C3 monitoring is recommended to adjust the dosage of MZR [12], the Cmax is known to be delayed in patients on dialysis, and the concentration has been shown to be maintained until the next dialysis session [2, 3]. When the serum concentration of MZR is 1.0 μg/mL, activation of lymphocytes is suppressed by approximately 50%, and when the concentration is 0.8–3.0 μg/mL, MZR binds to 14-3-3 protein and activates a steroid receptor [7, 13]. Adverse events, such as liver dysfunction or pancytopenia, increase when blood levels exceed 5 μg/mL [8, 13].

There have been a few reports in which MZR was administered to AAV patients on hemodialysis, and we found two relevant reports. One case was received MZR (75 mg) after each dialysis session as remission maintenance therapy for AAV; the other received MZR (50 mg) after each dialysis session at the time of a relapse of AAV. In those cases, the Cmax of MZR was 0.53–1.84 μg/mL [2, 3]. In the present case, the administration of MZR (50 mg) after each dialysis session with the Cmax of MZR controlled at 1.49 μg/mL was effective for AAV with ANCA and anti-GBM antibody double positivity and no severe adverse events were observed.
